# Fungating Breast Wound: A Rare Manifestation of Primary Breast Leiomyosarcoma

**DOI:** 10.7759/cureus.33398

**Published:** 2023-01-05

**Authors:** Catarina Osório, Egon F Rodrigues, Mariana Santos, Teresa Santos, Mário Nora

**Affiliations:** 1 General Surgery, Centro Hospitalar de Entre Douro e Vouga, Santa Maria da Feira, PRT

**Keywords:** best supportive care, metastatic, breast cancer, breast leiomyosarcoma, fungating breast wound

## Abstract

Leiomyosarcomas are the least frequent primary breast sarcomas, making it an extraordinarily rare malignancy. The clinical manifestation of this entity as a fungating breast wound is, on its own, highly unusual in developed nations, mainly due to the improvement of worldwide screening programs and easier access to health care. Management of this breast wound remains challenging, and an accurate histopathological diagnosis is essential for a proper treatment plan. Thus, we present this rare case of metastatic breast leiomyosarcoma to contribute to the scarce literature regarding this disease.

## Introduction

Breast sarcomas, first described by Schmidt in 1887, are a rare type of nonepithelial malignancies, histologically heterogeneous, that arise from the mesenchymal tissue of the breast. They account for less than 1% of all primary breast tumors and include malignant phyllode tumors, post-radiation sarcomas, and different histopathological subtypes of soft tissue sarcomas [1-4].

Leiomyosarcoma is the least common subtype, representing 5 to 10% of breast sarcomas. Due to its rarity, only a few cases are described in the literature. Therefore, this type of tumor's diagnostic and therapeutic approaches are highly heterogeneous to a need for more specific treatment strategies and guidelines [5,6].

The clinical presentation of a primary breast leiomyosarcoma as a fungating breast wound has not yet been reported in the literature. Fungating breast wound is a rare manifestation of locally advanced breast cancers that occurs due to the infiltration of malignant cells in the skin, causing loss of skin integrity, inflammation, and infection. The optimal management of these wounds remains challenging, and the life expectancy of these patients is limited [6].

The authors report a case of a metastatic primary breast leiomyosarcoma manifesting as a fungating breast wound in a pre-menopausal female patient.

## Case presentation

**Figure 1:**
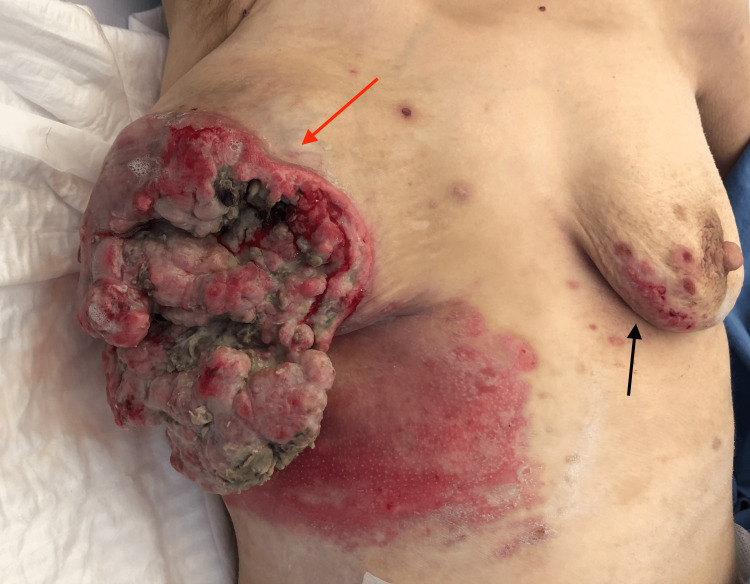
Fungating breast wound Large fungating mass of the right breast with massive ulceration of the skin (red arrow) and small skin lesions compatible with cutaneous metastasis in the contralateral breast  (black arrow).

**Figure 2:**
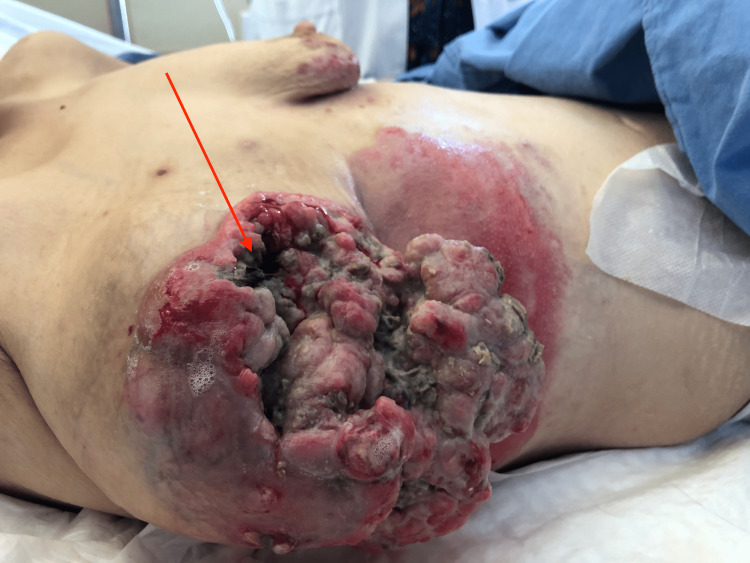
Large fungating breast wound with bacterial over-infection and necrosis Arrow identifying necrotic tissue in the breast wound.

A 39-year-old female patient was referred to our hospital due to severe anorexia, weight loss (20 kg in one year), weakness, and prostration. After careful interrogation, she revealed the presence of a bulky, fast-growing breast mass with six-month evolution. Upon physical examination, the patient presented with paleness, severe cachexia, and hypotension upon admission. During clinical breast examination, we identified a 20 x 20cm fungating mass in the right breast with massive ulceration of the skin and small skin lesions compatible with cutaneous metastasis in the contralateral breast (Figure 1, 2).

**Figure 3:**
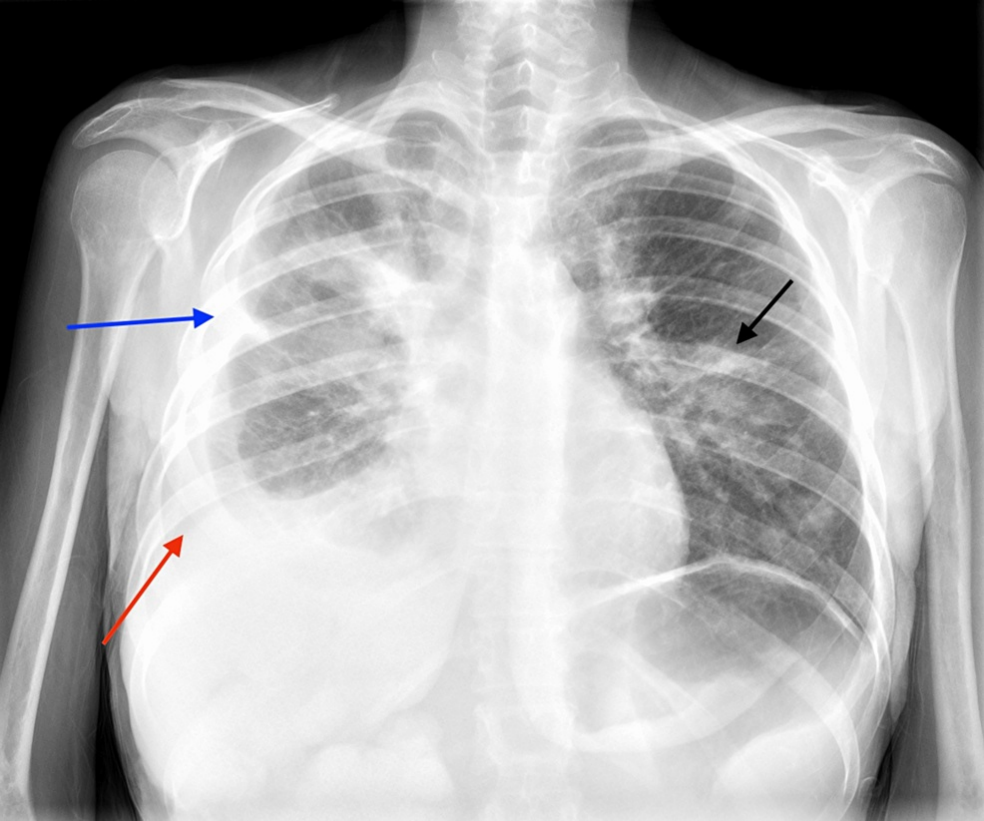
Chest radiogram at admission Chest radiogram revealing a right pleural effusion (red arrow) with multiple bilateral pulmonary nodules (black arrow) and pleural retraction (blue arrow) in the upper third of the right lung.

Laboratory tests showed severe anemia (5,1 g/dL), leukocytosis (60,700/mm^3^), elevated levels of C-reactive protein (331 mg/L), procalcitonin (3,52 ng/mL) and altered International Normalized Ratio (INR) of 2,1. The chest radiogram revealed a right pleural effusion with multiple bilateral pulmonary nodules and pleural retraction in the upper third of the right lung (Figure 3).

The patient was admitted for a definite diagnosis, staging, and treatment of bacterial over-infection of the fungating breast neoplasm. The patient was given broad-spectrum antibiotics and transfused packed red cells. We began dressing care of the fungating wound with topical metronidazole.

**Figure 4:**
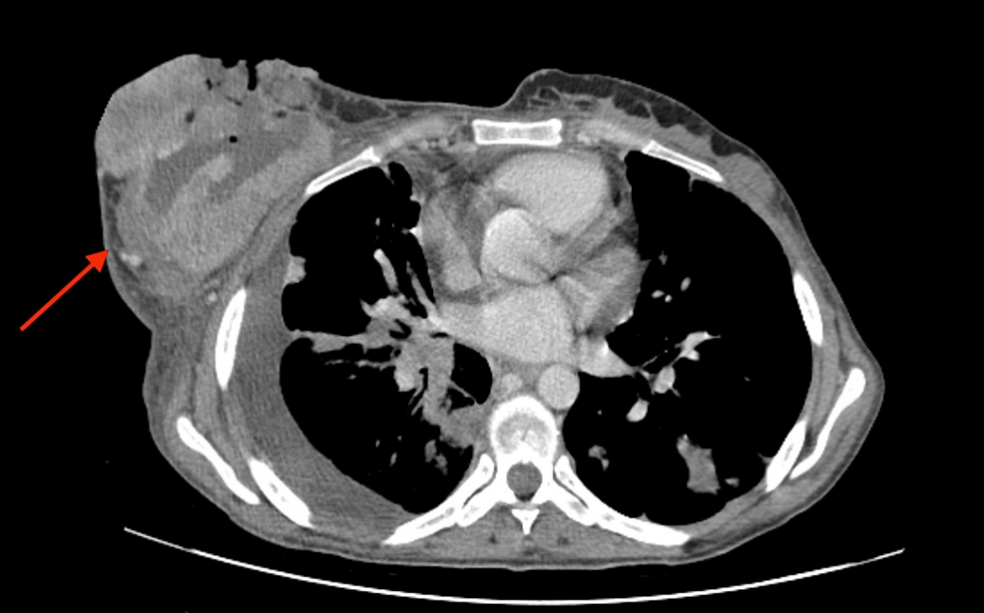
Contrast-enhanced thoracoabdominal CT A large neoformative lesion in the right breast (red arrow), ulcerated, with air-fluid levels inside.

**Figure 5:**
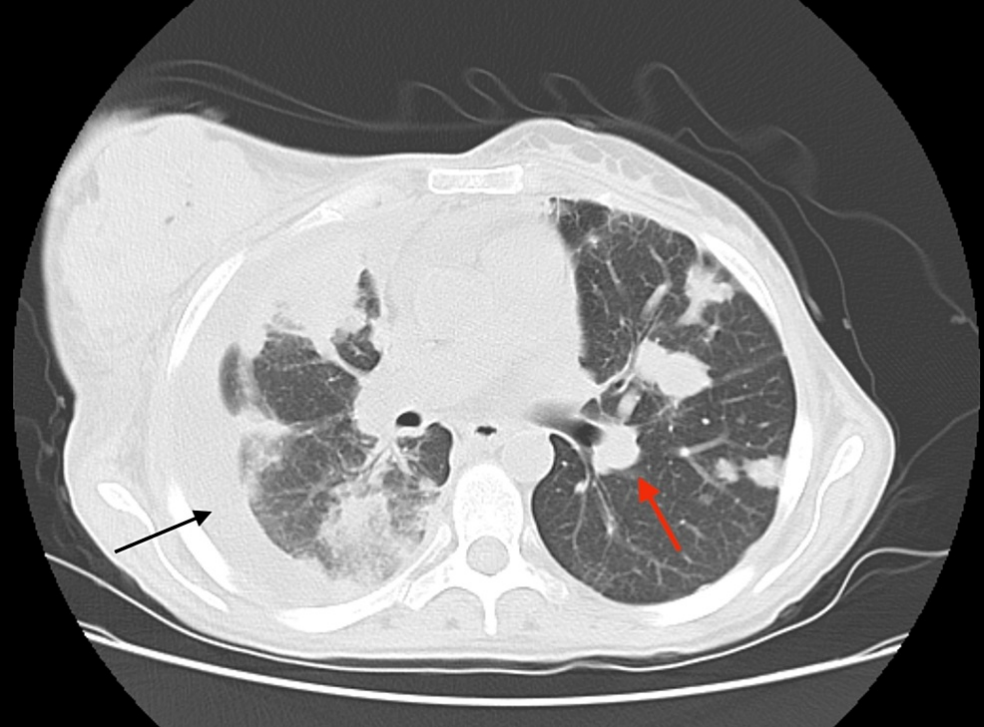
Pleural carcinomatosis and pulmonary metastases Contrast-enhanced thoracic CT showing a loculated right pleural effusion with circumferential irregular pleural thickening (black arrow) and multiple pulmonary nodular lesions with spiculated contours (red arrow).

A contrast-enhanced computed tomographic (CT) of the thorax, abdomen, and pelvis was performed for staging, which demonstrated a large neoformative lesion in the right breast (with approximately 15 cm), ulcerated, with air-fluid levels inside and enlarged ipsilateral axillary lymph nodes (Figure 4). A loculated right pleural effusion with circumferential irregular pleural thickening due to pleural carcinomatosis and multiple pulmonary nodular lesions with spiculated contours in favor of multiple pulmonary metastases (Figure 5) were identified. The CT scan showed bone metastases in the pelvis.

An ultrasound-guided core needle biopsy of the breast mass was conducted for a definite diagnosis. The histopathological examination revealed a spindle-cell neoplasm with moderated nuclear atypia and rare mitosis figures. Tumor cells were strongly positive for smooth muscle actin and vimentin and lacked expression of cytokeratin 7, CD34, Cam 5.2, and protein S-100. The ultrasound of the contralateral breast was clear, except for localized skin thickening suggestive of cutaneous skin metastasis. According to the histopathological and immunohistochemical findings, the tumor was classified as a leiomyosarcoma of the breast.

Staging revealed massive dissemination of the leiomyosarcoma with lung, pleural, cutaneous, and bone metastasis. After a discussion of the case with a tertiary center with a Sarcoma Treatment Unit, the patient was referred to palliative care due to the extent of the disease and died one month after the diagnosis.

## Discussion

Leiomyosarcomas are a rare subtype of breast sarcomas, and its exact origin is debated. They belong to a subgroup of spindle cell tumors of the breast that arise from the smooth-muscle cells lining blood vessels or stromal mesenchymal cells. Thus, they are most likely to develop near the areola from this anatomic region's blood vessels and musculature [4,7-8].

The clinical presentation of breast leiomyosarcomas is often a slow-growing large palpable mass, painless, firm, and lobulated, typically found in postmenopausal women [1,9]. In this case, the primary clinical presentation was a large fungating breast wound in a pre-menopausal patient. To the best of our knowledge, our patient is one of the rare cases of primary breast leiomyosarcoma to be diagnosed with this kind of wound and in such an advanced stage of the disease. This unique form of presentation of breast tumors is sporadic in developed nations due to screening programs, better awareness of breast diseases, and easy access to health care. The clinical presentation of a fungating breast wound is associated with a worse prognosis. The lack of evidence-based management guidelines for this kind of wound makes its management a clinical challenge. Although the optimal treatment approach is unknown, it should involve a multidisciplinary team. Given the short life expectancy of these patients, the treatment strategy must be based on loco-regional control to help alleviate pain and bleeding and improve quality of life. Furthermore, these women have numerous physical and psychosocial side effects due to the complications of the fungating breast wounds. Providing social and psychological support is essential to treatment [6,10]. 

Imaging modalities are neither sensitive nor specific, and there are no imagiological characteristics of leiomyosarcomas. Thus, these are often mistaken for benign lesions. Definitive diagnosis is only possible with histological examination and immunohistochemical analysis after core biopsy. Leiomyosarcoma presents prominent cell atypia, atypical mitosis, vascular invasion, and necrosis. Immunohistochemical staining is essential for the differential diagnosis between leiomyosarcomas and other soft tissue sarcomas: these tumors are usually positive for vimentin and smooth muscle actin and negative for S-100, cytokeratins and epithelial markers [4-8].

Regarding the treatment strategy, given the limited experience of single institutions and the small number of case reports published, there are no specific recommendations regarding surgery and medical treatments for this entity. Therefore, data has been extrapolated from studies of other soft tissue sarcomas, case reports, and small case series to define the best therapeutic approach for breast leiomyosarcomas. [7]

At this time, the cornerstone of treatment for early-stage disease is surgical excision with wide margins. Mastectomy is usually required to achieve local control in patients with large primary tumors [3]. Recommendations on surgical resection margins are scarce because of their low incidence. However, data from systematic reviews recommend a 3 cm margin as adequate. There have been no reported cases of lymph node metastasis; therefore, axillary node dissection affords no benefit except when the diagnosis cannot be achieved before surgery. The benefits of adjuvant chemotherapy and radiotherapy are still being determined. Postoperative radiotherapy may be indicated, mainly when wide excision is impossible [9,11].

Dissemination of breast leiomyosarcomas is mainly hematogenous, so that these tumors can present with distant metastases. Metastases are more often in the lungs and bones. In patients with metastatic disease, palliative chemotherapy or palliative surgery can be offered to delay disease progression and control local complications [5,7]. In this case, the presence of widely disseminated disease and a large fungating breast wound led us to refer the patient to the best supportive care.

The prognosis of breast leiomyosarcomas is usually optimistic relative to that of other breast sarcomas. The prognostic factors are not fully known, although some studies consider the degree of local invasion, cellular atypia, and mitotic rate related to patient prognosis. Some reports also consider tumor size as an important prognostic factor. Because of the prolonged risk of recurrence, close follow-up may be considered [5,9,12].

## Conclusions

Primary breast leiomyosarcoma is a diagnostic challenge to clinicians considering the rarity of this entity and the limited data available. Reporting on unique cases of these types of tumors is essential to guide treatment decisions. The natural history of the disease and the response of the tumor to different therapeutic modalities have not been established. Surgical treatment remains the best approach, but general agreement on the treatment of metastatic disease is still lacking and is defined on a single-patient basis. In this case, managing fungating breast wound was uniquely tricky. The optimal management of this clinical presentation of breast tumors should be focused on prevention by increasing public awareness and education for early diagnosis.
